# Analysis of COVID-19 in Professionals Working in Geriatric Environment: Multicenter Prospective Study

**DOI:** 10.3390/ijerph18189735

**Published:** 2021-09-15

**Authors:** Anca-Maria Mihai, Jérémy Barben, Mélanie Dipanda, Jérémie Vovelle, Valentine Nuss, Camille Baudin-Senegas, Alain Putot, Patrick Manckoundia

**Affiliations:** 1 “Pôle Personnes Âgées”, Hospital of Champmaillot, University Hospital, 21079 Dijon, France; anca-maria.mihai@chu-dijon.fr (A.-M.M.); jeremy.barben@chu-dijon.fr (J.B.); melanie.dipanda@chu-dijon.fr (M.D.); jeremie.vovelle@chu-dijon.fr (J.V.); valentine.nuss@chu-dijon.fr (V.N.); camille.senegas@chu-dijon.fr (C.B.-S.); alain.putot@chu-dijon.fr (A.P.); 2 INSERM U-1093, Cognition, Action and Sensorimotor Plasticity, University of Burgundy Franche-Comté, 21000 Dijon, France

**Keywords:** geriatrics, healthcare workers, SARS-CoV-2, self-declaration survey

## Abstract

Healthcare workers (HCWs) are exposed to a higher risk of coronavirus disease (COVID-19) contamination. This prospective multicenter study describes the characteristics of HCWs tested for severe acute respiratory syndrome coronavirus 2 (SARS-CoV-2) while working in a geriatric environment. We also compared HCWs with a positive reverse transcription polymerase chain reaction (RTPCR) assay (RTPCR+ group) and those with a negative test result (RTPCR− group). Between 15/5/2020 and 15/9/2020, 258 HCWs, employed in the acute geriatric unit (AGU), geriatric rehabilitation unit (GRU) or nursing home of three hospitals in Burgundy (France) were invited to complete an online survey. Among the 171 respondents, 83 participants, with mean age 42 years and 87.9% female, were tested for SARS-CoV-2 infection. Among these 83 participants, COVID-19 was confirmed in 38 cases (RTPCR+ group) of which 36 were symptomatic, and the RTPCR assay was negative in 45 cases (RTPCR− group) of which 20 participants were symptomatic. A total of 22.9% (of 83) had comorbidities, 21.7% were active smokers, and 65.1% had received the flu vaccine. A total of 37.3% worked in AGU, 19.3% in GRU and 16.9% in nursing homes. The most common symptom described was headache (23.2%), followed by fatigue or cough (12.5% each), and fever or myalgia (10.7% each). There were more participants with normal body mass index (*p* = 0.03) in the RTPCR+ group. In contrast, there were more users of non-steroidal anti-inflammatory drugs (*p* = 0.01), active smokers (*p* = 0.03) and flu vaccinated (*p* = 0.01) in the RTPCR− group. No difference was found between the two groups for the type of work (*p* = 0.20 for physicians and *p* = 0.18 for nurses). However, acquiring COVID-19 was significantly associated with working in AGU (*p* < 0.001) and nursing homes (*p* = 0.001). There were significantly more users of surgical masks (*p* = 0.035) in the RTPCR+ group and more filtering facepiece-2 mask users (*p* = 0.016) in the RTPCR− group. Our results reflect the first six months of the COVID-19 pandemic in France. Further studies are needed to evaluate and track the risks and consequences of COVID-19 in HCWs.

## 1. Introduction

Since December 2019, the world has been grappling with the coronavirus disease (COVID-19) pandemic [1]. Currently (September 2021), more than 223.5 million cases of COVID-19 and 4.6 million associated deaths have been confirmed worldwide, which makes this pandemic one of the most severe to have affected humanity [2]. COVID-19 mainly affects the lungs, causing an interstitial infection, and severe acute respiratory distress syndrome occurs in up to 25% of cases, depending on the risk factors associated [3]. Studies from China and Italy suggest a case-fatality ratio of 2.3% [4]. About 17% to 25% of individuals with COVID-19 will remain asymptomatic, and the infection is mild to moderate in 81% of cases [5].

The prevalence and morbidity of COVID-19 continues to increase, due in large part to community contamination facilitated by asymptomatic individuals. Healthcare workers (HCWs) have a high risk of contracting COVID-19 due to direct contact with the secretions of infected patients [6]. Data from the United States and Great Britain suggest that HCWs are 3.4 times more at risk of contamination than the general population [7]. In France, the national census of COVID-19 cases suggests that between 1 March 2020 and 8 March 2021, more than 59,000 HCWs were infected with severe acute respiratory syndrome coronavirus 2 (SARS-CoV-2) [8]. While many studies have described COVID-19 in the general population, there is currently little available data regarding this disease, i.e., clinical manifestations or contamination context, for example, in HCWs working in a geriatric environment. In addition, HCWs on the front line, particularly in the geriatric environment, had very little information about COVID-19 during the first wave.

This study aimed to describe the sociodemographic and clinical characteristics of HCWs tested for SARS-CoV-2 while working in a geriatric environment. We also sought to analyze the generally described risks and protective factors for COVID-19 in this same population of HCWs through some of the questions in the survey. Finally, we compared HCWs with positive reverse transcription polymerase chain reaction (RT-PCR) assay to those with a negative result.

## 2. Methods

### 2.1. Design

We conducted an epidemiological, descriptive, self-reporting, multicenter study using data obtained via an anonymous digital survey between 15 May and 15 September 2020, in three hospitals in Burgundy (France). This study was conducted in accordance with the Declaration of Helsinki and French national standards. The Ethics Committee of our institution was consulted. It approved this study, which had no impact on participant management.

### 2.2. Population Selection

The target population consisted of HCWs, including physicians, nurses, assistant nurses, physiotherapists, occupational therapists, psychologists, dieticians, administrative staff or others. The participants worked in one or multiple types of geriatric establishments: 3 acute geriatric units (AGU), which at the time were not COVID-19 units, 2 geriatric rehabilitation units (GRU) and 5 nursing homes.

The only exclusion criterion was individual refusal.

The invitation to participate in the survey was sent to the professional e-mail of potential participants. In total, 258 invitations were sent. At the beginning of the survey, participants were required to consent to the use of their anonymized information for research purposes.

Depending on the result of the RTPCR test, two groups were constituted: the RTPCR+ group, including individuals with a positive test result, and the RTPCR− group, including HCWs with a negative test result. We chose to include HCWs who tested negative for COVID-19 because, although the RTPCR test is widely considered as the gold standard for COVID-19 diagnosis (high sensitivity and specificity) [9], false negatives are possible depending on when the sample is collected (latency or incubation) and the quality of the biological sample. Thus, COVID-19 could not be completely ruled out in symptomatic HCWs with a negative RTPCR result.

### 2.3. Diagnosis of Prevalent SARS-CoV-2 Infection

A positive case of COVID-19 was defined as a symptomatic or asymptomatic HCW testing positive in the RTPCR assay performed on a nasopharyngeal swab for SARS-CoV-2 detection [10]. Depending on the type of geriatric establishment, local guidelines were applied, and swab tests were either conducted systematically or only in the case of symptomatic HCWs.

### 2.4. Data Collection

The survey was conducted using a free online software program named Drag’n Survey. It consisted of 35 questions related to sociodemographic characteristics (age, sex, type of household and comorbidities), work description, clinical manifestation and complications of the SARS-CoV-2 infection, risk behaviors, and questions related to protective personal hygiene measures (Table 1). The survey was available for a period of 45 days for each of the 3 hospitals, and two reminders were sent during this period (at day 15 and day 30). The data were directly collected in Excel format using XL STAT software (Addinsoft, Paris, France).

### 2.5. Statistical Analysis

Continuous variables were described as means, while categorical variables were described as numbers and percentages. The RTPCR+ group and the RTPCR− group were compared using Chi-squared test or *T*-student test in univariate analysis. Statistical significance was set at *p* < 0.05. XL STAT software was used to conduct all statistical analyses on an Excel spreadsheet.

## 3. Results

**Figure 1:**
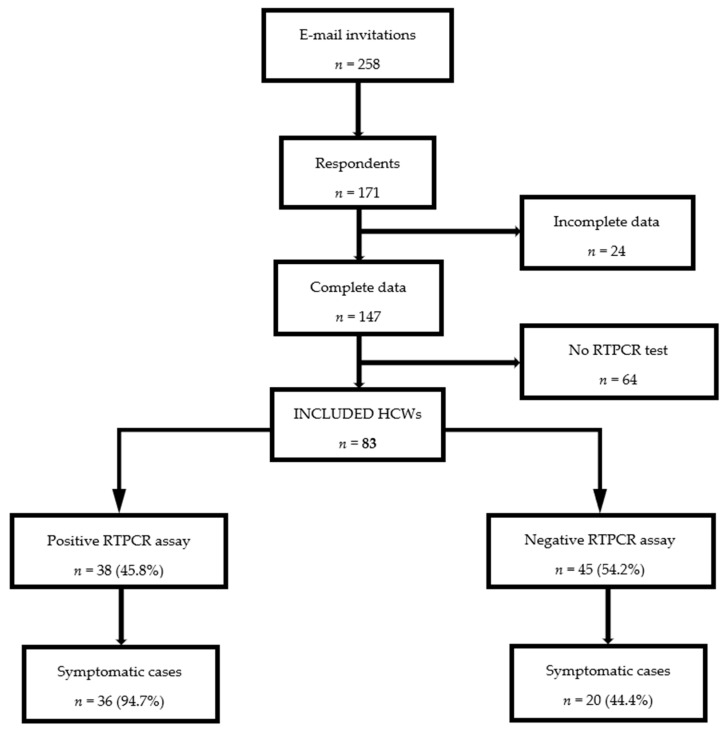
Study flow chart (RTPCR: reverse transcription polymerase chain reaction, HCWs: healthcare workers).

During the study period, 171 (66.3%) of the 258 invited HCWs answered the survey. Twenty-four were excluded due to incomplete data and 64 because they could not provide a COVID-19 RTPCR test. Among the 64, 31 (48.4%) had symptoms compatible with COVID-19. Thus, 83 (56.5%) HCWs were tested by RTPCR. Among these 83 HCWs, SARS-CoV-2 infection was confirmed in 38 cases (45.8%) of which 36 (94.7%) were symptomatic. The RTPCR assay was negative for 45 participants (54.2%) of which 20 (44.4%) were symptomatic. Figure 1 reports the flow chart.

### 3.1. Sociodemographic and Medical Data

The mean age of the 83 participants was 42.4 ± 10.5 years and 87.9% were females. Furthermore, 84.3% lived in a household with multiple inhabitants and 48.2% with children. Body mass index (BMI) was normal in 57.8% of cases and 22.9% presented comorbidities, including cardiovascular disease in 9.6% of cases, auto-immune diseases in 4.8% (4) of cases, and lung diseases in 3.6% of cases. Furthermore, 22.9% had consumed topical or oral non-steroidal anti-inflammatory drugs (NSAIDs) in the last 6 months and 8.4% topical or oral corticosteroids. Finally, 21.7% were active smokers and, 65.1% received the flu vaccine during the 2019–2020 flu season. All these data are displayed in Table 2.

### 3.2. Work-Related Characteristics

The mean time working in a geriatric service was 10.7 ± 9.7 years. Furthermore, 85.5% of individuals worked full-time hours and 8.4% in night shifts. Regarding the type of work, 31.3% were physicians, 27.7% were nurses, and 22.9% were assistant nurses. Overall, 37.3% worked in an AGU, 19.3% in a GRU and 16.9% in a nursing home. Additional data are provided in Table 3.

### 3.3. Clinical Presentation

Among the 83 participants, 67.5% had symptoms compatible with a SARS-CoV-2 infection and in 43.3% of cases the disease was confirmed by RTPCR. The first symptom described was headache (23.2% of cases), followed by fatigue or cough (12.5% each), then fever or myalgia (10.7% each). The most common presentation was the association of fatigue (56.6%), headache (48.2%), ageusia or anosmia (32.5% each) and fever (24.1%). The mean duration of symptoms was 17.7 ± 18.3 days. In total, 59% (49) of all participants went on sick leave. The average duration of the sick leave was 14.9 ± 10.5 days. In 39.8% of cases, there were residual symptoms. The most frequent were asthenia (48.5%) and dyspnea (39.4%). There was no declared hospitalization related to COVID-19. Further information about clinical presentation is presented in Table 4 and Table 5.

### 3.4. Use of Personal Protective Equipment against COVID-19

In total, 79.5% (66) of participants used personal protective equipment (PPE) as a precaution against droplet and contact contamination (medical masks, gowns, eye protection). The most available PPE was the surgical mask, used in 75.9% of cases, followed by filtering facepiece-2 (FFP2) mask or N95 respirator in 34.9% of cases, and medical gowns in 8.4% of cases. Furthermore, 91.5% (76) of participants respected the protective personal hygiene measures in the professional environment: 87.9% (73) maintained a distance of one meter and 54.2% declared that they kept their surgical mask on during breaks with colleagues. These data are given in Table 6.

The most common explanation for COVID-19 contamination was hospital exposure, which was reported in 97.4% (37) of cases. Among these cases, 71.05% of contaminations were due to exposure to patients and 26.3% from colleagues who tested positive for COVID-19. Among those who acquired the infection, 81.6% (31) confirmed the presence of COVID-19 in their workplace before their infection.

### 3.5. Comparison between RTPCR+ and RTPCR− Groups

There was no significant difference between the RTPCR+ and RTPCR− groups for age (*p* = 0.3), sex (*p* = 0.28), type of household (*p* = 0.73), history of cardiovascular (*p* = 0.42), autoimmune (*p* = 0.13) or pulmonary (*p* = 0.33) diseases, or consumption of corticosteroids (*p* = 0.53) (Table 2). However, there were significantly more participants with normal BMI (*p* = 0.03) in the RTPCR+ group. In contrast, there were more users of NSAIDs (*p* = 0.01), active smokers (*p* = 0.03) and individuals vaccinated against flu (*p* = 0.01) in the RTPCR− group (Table 2).

No significant difference was found between the two groups concerning the duration of work in geriatric environment (*p* = 0.06), the time of work (*p* = 0.71) or the type of work (*p* = 0.20 for physicians *p* = 0.18 for nurses, *p* = 0.38 for assistant nurses, and *p* = 0.88 for administrative staff) (Table 3). However, a significant association was found between acquiring COVID-19 and working in AGU (*p* < 0.001) or nursing homes (*p* = 0.001) (Table 3).

Compared to the RTPCR− group, participants of the RTPCR+ group presented significantly longer duration of symptoms (21.7 ± 20.9 days vs. 10.4 ± 9.1 days, *p* = 0.007) and more residual symptoms (68.4% vs. 15.6%, *p* = 0.003) (Table 4). A total of 94.7% (36) of HCWs in the RTPCR+ group (all symptomatic participants) were given sick leave vs. 28.9% (13) in the RTPCR− group (*p* < 0.001). There was no difference in the mean duration of sick leave (16.2 ± 9.6 vs. 11.2 ± 11.8, *p* = 0.19) (Table 4). The most common clinical presentation of COVID-19 in the RTPCR+ group was headache (19.4%), followed by fatigue or myalgia (both 13.9%) and fever or anosmia (both 8.3%) (Table 3). There were also significantly more individuals with asthenia (*p* < 0.001), headache (*p* = 0.004), loss of taste (*p* < 0.001), anosmia (*p* < 0.001), anorexia (*p* = 0.003) and dyspnea (*p* = 0.001) in the RTPCR+ group compared to the RTPCR− group (Table 5).

In total, 73.7% of participants of the RTPCR+ group had access to PPE versus 84.4% in the RTPCR− group, but the difference was not significant (*p* = 0.23). There were significantly more users of surgical masks as part of protective personal hygiene measures in the RTPCR+ group (*p* = 0.049) and during working hours (*p* = 0.03), and significantly more FFP2 mask users as part of PPE in the RTPCR− group (*p* = 0.016). There was not a significant difference between groups regarding the use of FFP2 mask as part of protective personal hygiene measures (*p* = 0.62) (Table 6).

## 4. Discussion

During the first wave, the COVID-19 pandemic severely affected older adults and, therefore, impacted the organization and working conditions in geriatric facilities. Consequently, we focused this study on HCWs working in geriatric environments. In the literature, there are some studies with similar objectives, but few of these studies included all types of hospital professionals (including administrative staff) [11,12]. In addition, the few studies on COVID-19 in HCWs in the geriatric environment did not have the same objective as our paper [13]. Indeed, we were interested in the socio-demographic characteristics, clinical features and the concerns of HCWs working in the geriatric environment regarding COVID-19; hence, the added value of our study is apparent.

In our study, only 56.5% (83) of the survey respondents were tested for COVID-19 by RTPCR. As for the 64 HCWs excluded from the study due to the absence of RTPCR test, almost half (48.4%; 31) were symptomatic. Considering the dramatic increase in COVID-19 cases in the general population and hospital staff, it may seem surprising that RTPCR were not performed more systematically in HCWs, whether or not they were symptomatic.

The mean age of the total population of HCWs was 42 years, and 42.1% of participants were registered nurses. This rate is similar to that found (38.6%) in a systematic review on COVID-19 in HCWs worldwide [14]. The same review found that the second category of HCWs most affected by COVID-19 were physicians, as in our study [14].

We found that 63.2% of subjects in RTPCR+ group worked in an AGU (non-COVID unit). This is consistent with data from a study conducted in the staff of two Paris hospitals, which showed that 70% of the infected participants worked in an acute medicine department [15]. This could be because there are more aerosol-generating gestures in this type of medical department (for instance insertion of nasogastric tubes, tracheal aspiration, and high concentration mask oxygen therapy), as well as close and frequent contact with the patient, leading to prolonged cumulative exposure periods [16].

In our study, no association was found between the COVID-19 risk and the presence of obesity (*p* = 0.42), cardiovascular diseases (*p* = 0.42), type 2 diabetes (*p* = 0.63), lung diseases (*p* = 0.33) or an autoimmune disease (*p* = 0.13). This could be explained by the rather young mean age of our participants (42 years) compared to the mean age of the general population infected by SARS-CoV-2 (62 years) in the same period [17]. Another possible explanation is the fact that younger participants could have comorbidities that have not yet been diagnosed. We found that there were significantly fewer flu vaccinated HCWs in the RTPCR+ group compared to RTPCR− group (19% vs. 35%, *p* =0.01). This is consistent with other studies having found that people who were vaccinated against the influenza virus have a reduced risk of SARS-CoV-2 infection (up to 24%) and fewer severe forms requiring hospitalization in an intensive care unit [18].

There were fewer active smokers in the RTPCR+ group than in the RTPCR− group (10.5% vs. 31.1%, *p* = 0.03). Since the beginning of COVID-19 pandemic, a “smoker’s paradox” has been described in several studies because of an under-representation of active smokers among COVID-19 patients [19,20]. Some of the plausible mechanisms that were suggested included the anti-inflammatory effect of nicotine, the reduced risk of cytokine shock because of a decreased immune response, and the increased level of nitric oxide in the smoker’s airways that could inhibit the replication of the coronavirus [20]. A large English study has shown that smoking was associated with a higher risk of COVID-19 mortality after age and sex adjustment [21]. However, paradoxically, after adjustment for other addition co-variables, the same study found an association between smoking and a decrease in mortality risk [21]. A meta-analysis including 19 studies and involving 11,590 COVID-19 patients found a significant association between smoking and worsening of the infection, suggesting that quality limitations of some studies may underestimate the effect of smoking [22]. At present, the data are not clear enough to confirm the impact of smoking on COVID-19.

We found significantly fewer consumers of NSAIDs in the RTPCR+ group than in the RTPCR− group (10.5% vs. 33.3%, *p* = 0.01). Although a few studies suggest that NSAIDs used as early as possible in the course of COVID-19 could prevent the infection from worsening or even reverse the associated lymphopenia [23], the protective effect of this medication remains controversial.

In our survey, most participants had no specific initial symptoms. However, the clinical presentation of COVID-19 combines a collection of representative signs including ageusia, anosmia, anorexia, asthenia, headache and shortness of breath, which were significantly more frequent in the RTPCR+ group than in the RTPCR− group [all *p* < 0.001, except for anorexia (*p* = 0.003) and headache (*p* = 0.004)]. In the RTPCR+ group, the mean duration of symptoms was 22 days. All the symptomatic individuals from this group were off work for at least 14 days (mean 16.2 days), which was in line with the French guidelines during the target period [24].

Our findings indicate that 71.05% of hospital-acquired infections resulted from contact with COVID-19 patients, whereas 26.3% of participants believed that they had been contaminated after contact with an infected colleague, particularly during “break times”. All of the HCWs in this cohort had mild symptoms that did not require hospitalization. This could be due to the younger mean age of the RTPCR+ group (41 years), as suggested by a Chinese study that reported that age was inversely related to the severity of the SARS-CoV-2 infection [25].

During the survey, 79.5% of participants declared having had access to PPE (masks, gown, apron). However, in most cases, access to this equipment was limited in time since it was introduced gradually and, sometimes, fairly late in relation to the spread of the virus in hospitals and in the general population. In our study, the use of FFP2 masks (*p* = 0.016) reduced the risk of SARS-CoV-2 infection during medical and paramedical procedures, but we were unable to find the same link for those who used surgical masks during working hours. Indeed, there were significantly more HCWs wearing surgical masks in the RTPCR+ group either during working hours (*p =* 0.035) or during breaks (*p* = 0.049). This may be explained by the misuse of protective equipment when surgical masks were rationed following the international shortage of masks at the beginning of the first COVID-19 wave. There is little evidence of a difference in effectiveness between surgical masks and FFP2 masks in preventing SARS-CoV-2 infection [26].

During the first wave of COVID-19 (March–April 2020), little was known about the spread or carriage of the virus and aerosol-generating procedures. In France and many other countries, the protocols concerning PPE were not well defined and the protocols often differed from hospital to hospital. During the survey, multiple participants made comments reflecting the psychological and physical distress induced by the lack of communication, lack of clear and coherent information, and insufficient support.

This study has some limitations. First, the declarative nature of the survey may call into question the veracity of certain information and provide an inclusion bias (it is likely that symptomatic persons were more willing to respond to the survey. In addition, while we wanted to represent all types of HCWs, some responded less, likely due to the fact that some people did not have access to internet, as the survey was available online only). The second limitation is the relatively small number of useable questionnaires and the possible underestimated number of infected HCWs (due to under-reporting and lack of RTPCR tests available in different hospital departments). Third, in order to maximize the response rate and the number of fully completed surveys, we limited the number of questions. We therefore decided not to collect complementary information such as the type of NSAIDs and their duration of use, the duration of smoking, evaluation of accessibility to PPE and the existence or not of regular training in the use of PPE.

## 5. Conclusions

The rapid international spread of SARS-CoV-2 put intense pressure on the health care system and particularly frontline HCWs, who were exposed to a higher risk of COVID-19 contamination at work. Our results reflect the first six months of the COVID-19 pandemic in France, although representing a smaller scale and the early interaction of HCWs with a previously unknown virus. We found that amongst HCWs who tested positive for SARS-CoV-2, those working in an AGU (non-COVID unit) or in nursing homes were at a higher risk of contracting COVID-19. The highest risk was among nurses exposed to COVID-19-positive patients. Flu-vaccinated HCWs were less likely to contract SARS-CoV-2 infection. Active smoking and consuming NSAIDs seemed to have a similar effect, but more data is needed to gain an understanding of their mechanism of action in SARS-CoV-2. There was an association between wearing an FFP2 mask during working hours and a low risk of COVID-19 infection. More information is needed to assess the peculiarities of COVID-19 contamination in high-risk populations such as HCWs, especially those working in geriatric facilities. It will also be important to continue advancing our understanding of the diagnostic and managerial difficulties that occurred in the medical units most severely affected by the pandemic.

## Figures and Tables

**Table 1:** Questionnaire of the survey.

No.	Question	Response
1	How old are you?	Value:
2	What is your sex?	□Woman □Man
3	What is your body mass index?	Value:
4	What is your smoking status?	□Non-smoker □Smoker
5	Did you receive a flu vaccine in 2019–2020?	□Yes □No
6	Is there more than one person living in your home?	□Yes □No
7	Do you live in a household with children?	□Yes □No
8	Do you live in a household with older person(s) (≥75 years)?	□Yes □No
9	What is your work in the unit/department?	□Physician □Nurse □Assistant nurses □Physiotherapist □Occupational therapist □Dietitian □Psychologist □Member of administrative staff □Other
10	In what unit/department do you work?	□AGU □GRU □NH □No fixed unit
11	Do you work full time?	□Yes □No
12	Are you occupying a night shift?	□Yes □No
13	How long have you been working in this hospital (years)?	Value:
14	Do you suffer from a chronic illness?	□Yes □No
15	In the last 6 months have you used (topically or orally) NSAIDs or corticosteroids?	□Yes □No
16	In the last 6 months have you had symptoms consistent with COVID-19?	□Yes □No
17	What symptoms have you had?	□Fever □Cough □Dyspnoea □Asthenia □Myalgia □Sputum production □Headache □Sore Throat □Nasal obstruction □Diarrhea □ Anorexia □Nausea □Anosmia □Ageusia □Another
18	What was the first symptom?	□Fever □Cough □Dyspnea □Asthenia □Myalgia □Sputum production □Headache □Sore Throat □Nasal obstruction □Diarrhea □Anorexia □Nausea □Anosmia □Ageusia □Another
19	How long did the symptoms last (days)?	Value:
20	Were you hospitalized because of these symptoms?	□Yes □No
21	Do you have residual symptoms? If yes, what are those symptoms?	□Yes □No Response:
22	Have you been tested for SARS-CoV-2 infection by nasopharyngeal RTPCR test?	□Yes □No
23	If you were tested for COVID-19, what was the result of the RTPCR test?	□Negative □Positive
24	If you were tested, was the test performed before or after the start of symptoms?	□Yes □No
25	Did you go on sick leave?If yes, how long was your sick leave (days)?	□Yes □No Value:
26	Did you have a chest computed tomography scan?	□Yes □No
27	If yes, were the lung lesions compatible with a SARS-CoV-2 infection?	□Yes □No
28	What type of acquired infection do you suspect?	Response:
29	Were there COVID-19 cases in your entourage?	□Yes □No
30	Did these cases occur before or after your viral infection?	□Yes □No
31	Since the beginning of the pandemic, did you have access to personal protective equipment?	□Yes □No
32	Since the beginning of the pandemic, when in close contact with a COVID-19 infected patient or resident, what PPE were you wearing?	Response:
33	Do you strictly respect the protective personal hygiene measures (regular hand washing, proper dressing and undressing before and after contact with infected patient/resident, proper mask use) during working hours?	□ Yes □No
34	During break hours, in the presence of one or more than one colleagues, what protective personal hygiene measures do you use?	Response:
35	Do you strictly respect the protective personal hygiene measures outside of working hours?	□Yes □No

AGU: geriatric unit, GRU: geriatric rehabilitation unit, NH: nursing home, NSAIDs: non-steroidal anti-inflammatory drugs, COVID-19: coronavirus disease, SARS-CoV-2: severe acute respiratory syndrome coronavirus 2, RTPCR: reverse transcription polymerase chain reaction.

**Table 2:** Sociodemographic and medical characteristics of healthcare workers and comparison between the RTPCR+ and RTPCR− groups.

Variable		Total (*n* = 83)	RTPCR+ Group (*n* = 38)	RTPCR− Group (*n* = 45)	*p* *
		Mean ± SD or % (*n*)	Mean ± SD or % (*n*)	Mean ± SD or % (*n*)	
Age (years)	Mean	42.4 ± 10.5	41 ± 9.4	43.5 ± 11.2	0.30
	≤25	3.6 (3)	5.3 (2)	2.2 (1)	0.52
	26–35	24.1 (20)	21.1 (8)	26.7 (12)	0.56
	36–45	34.9 (29)	42.1 (16)	28.9 (13)	0.22
	46–55	24.1 (20)	26.3 (10)	22.2 (10)	0.67
	56–65	13.3 (11)	5.3 (2)	20 (9)	0.055
Sex	Female	88 (73)	92.1 (35)	84.4 (38)	0.28
	Male	12 (10)	7.9 (3)	15.6 (7)	
Household type	Several persons	84.3 (70)	81.6 (31)	86.7 (39)	0.73
	With children	48.2 (40)	42.1 (16)	53.3 (24)	0.13
	With senior citizens	1.2 (1)	2.6 (1)	0 (0)	0.22
BMI	Mean ± SD	25.4 ± 5.2	24.6 ± 4.9	26.1 ± 5.3	0.15
	≤18.5	2.4 (2)	2.6 (1)	2.2 (1)	0.91
	18.5–24.9	57.8 (48)	71.1 (27)	46.7 (21)	0.03
	25–29.9	22.9 (19)	13.2 (5)	31.1 (14)	0.06
	≥30	16.9 (14)	13.2 (5)	20 (9)	0.42
Comorbidities	Cardiovascular diseases	9.6 (8)	5.3 (2)	13.3 (6)	0.42
	Autoimmune disease	4.8 (4)	7.9 (3)	2.2 (1)	0.13
	Lung diseases	3.6 (3)	5.3 (2)	2.2 (1)	0.33
	Type 2 diabetes	1.2 (1)	0 (0)	2.2 (1)	0.63
	Other	3.6 (3)	0 (0)	6.7 (3)	0.23
Drugs	NSAID	22.9 (19)	10.5 (4)	33.3 (15)	0.01
	Corticosteroids	8.4 (7)	10.5 (4)	6.7 (3)	0.53
Active smokers		21.7 (18)	10.5 (4)	31.1 (14)	0.03
Flu vaccine		65.1 (54)	50 (19)	77.8 (35)	0.01

N: number, RTPCR: reverse transcriptase polymerase chain reaction, SD: standard deviation, BMI: body mass index, NSAID: non-steroidal anti-inflammatory drugs. * Comparison between RTPCR+ and RTPCR− groups.

**Table 3:** Characteristics of work and comparison between the RTPCR+ and RTPCR− groups.

Variable		Total (*n* = 83)	RTPCR+ Group (*n* = 38)	RTPCR− Group (*n* = 45)	*p* *
		Mean ± SD or % (*n*)	Mean ± SD or % (*n*)	Mean ± SD or % (*n*)	
Duration of working in geriatric environment (years)		10.7 ± 9.7	8.6 ± 6.9	12.4 ± 12	0.06
Type of work schedule	Full-time	85.5 (71)	84.2 (32)	86.7 (39)	0.71
	Part-time	14.5 (12)	15.8 (6)	13.3 (6)	0.46
	Night shifts	8.4 (7)	10.5 (4)	6.7 (3)	0.56
Type of worker	Physicians	31.3 (26)	21.1 (8)	40 (18)	0.20
	Nurses	27.7 (23)	42.1 (16)	15.6 (7)	0.18
	Assistant nurses	22.9 (19)	18.4 (7)	26.7 (12)	0.38
	Administrative staff	15.7 (13)	13.2 (5)	17.8 (8)	0.88
	Physiotherapists	2.4 (2)	5.3 (2)	0 (0)	0.40
Department of work	Acute geriatric unit	37.3 (31)	63.2 (24)	15.6 (7)	<0.001
	Geriatric rehabilitation unit	19.3 (16)	15.8 (6)	22.2 (10)	0.47
	Rolling worker on several departments	18.1 (15)	18.4 (7)	17.8 (8)	0.93
	Nursing home	16.9 (14)	2.6 (1)	28.9 (13)	0.001
	Long-term care unit	2.4 (2)	0 (0)	4.4 (2)	0.53
	Other (mobile palliative care or geriatric team)	6 (5)	0 (0)	11.1 (5)	0.04

N: number, RTPCR: reverse transcriptase polymerase chain reaction, SD: standard deviation. * Comparison between RTPCR+ and RTPCR− groups.

**Table 4:** Clinical characteristics of COVID-19 in participants and comparison between the RTPCR+ and RTPCR− groups.

Variable		Total (*n* = 83)	RTPCR+ Group (*n* = 38)	RTPCR− Group (*n* = 45)	*p* *
		Mean ± SD or % (*n*)	Mean ± SD or % (*n*)	Mean ± SD or % (*n*)	
Duration of symptoms (days)		17.7 ± 18.3	21.7 ± 20.9	10.4 ± 9.1	0.007
Sick leave (number)		59 (49)	94.7 (36)	28.9 (13)	<0.001
Duration of sick leave (days)		14.9 ± 10.5	16.2 ± 9.6	11.2 ± 11.8	0.19
First symptoms	Headache	23.2 (13)	19.4 (7)	30 (6)	0.45
	Asthenia	12.5 (7)	13.9 (5)	10 (2)	0.67
	Cough	12.5 (7)	5.6 (2)	25 (5)	0.06
	Fever	10.7 (6)	8.3 (3)	15 (3)	0.51
	Myalgia	10.7 (6)	13.9 (5)	5 (1)	0.33
	Anosmia	5.4 (3)	8.3 (3)	0 (0)	0.24
	Total	67.5 (56)	94.7 (36)	44.4 (20)	0.006
Residual signs	Asthenia	48.5 (16)	50 (13)	42.8 (3)	0.14
	Dyspnea	39.4 (13)	38.5 (10)	42.8 (3)	0.37
	Anguish	12.1 (4)	11.5 (3)	14.3 (1)	0.34
	Thoracic angina	12.1 (4)	11.5 (3)	14.3 (1)	0.34
	Myalgia	12.1 (4)	11.5 (3)	14.3 (1)	0.34
	Cough	12.1 (4)	11.5 (3)	14.3 (1)	0.34
	Total	39.8 (33)	68.4 (26)	15.6 (7)	0.003

N: number, RTPCR: reverse transcriptase polymerase chain reaction, SD: standard deviation. * Comparison between RTPCR+ and RTPCR− groups.

**Table 5:** Most common clinical presentation and comparison between the RTPCR+ and RTPCR− groups.

Variable		Total (*n* = 83)	RTPCR+ Group (*n* = 38)	RTPCR− Group (*n* = 45)	p *
		% (*n*)	% (*n*)	% (*n*)	
Symptoms	Asthenia	56.6 (47)	81.6 (31)	35.6(16)	<0.001
	Headache	48.2 (40)	65.8 (25)	33.3 (15)	0.004
	Ageusia	32.5 (27)	68.4 (26)	2.2 (1)	<0.001
	Anosmia	32.5 (27)	65.8 (25)	4.4 (2)	<0.001
	Fever	24.1 (20)	31.6 (12)	17.8 (8)	0.16
	Chills	22.9 (19)	28.9 (11)	17.8 (8)	0.24
	Cough	19.3 (16)	21.1 (8)	17.8 (8)	0.71
	Myalgia	18.1 (15)	26.3 (10)	11.1 (5)	0.08
	Anorexia	15.7 (13)	28.9 (11)	4.4 (2)	0.003
	Dyspnea	13.3 (11)	23.7 (9)	4.4 (2)	0.001
	Rhinorrhea	10.8 (9)	7.9 (3)	13.3 (6)	0.46
	Insomnia	9.6 (8)	13.2 (5)	6.7 (3)	0.35
	Thoracic angina	7.2 (6)	13.2 (5)	2.2 (1)	0.07
	Tachycardia	6 (5)	5.3 (2)	6.7 (3)	0.82
	Palpitations	4.8 (4)	2.6 (1)	6.7 (3)	0.46
	Urticaria	1.2 (1)	2.6 (1)	0 (0)	0.46

N: number, RTPCR: reverse transcriptase polymerase chain reaction. * Comparison between RTPCR+ and RTPCR− groups.

**Table 6:** The use of personal protective equipment and comparison between the RTPCR+ and RTPCR− groups.

Variable			Total (*n* = 83)	RTPCR+ Group (*n* = 38)	RTPCR− Group (*n* = 45)	*p* *
			% (*n*	% (*n*)	% (*n*)	
PPE	PPE available		79.5 (66)	73.7 (28)	84.4 (38)	0.23
	Types of PPE	Surgical mask	75.9 (63)	86.8 (33)	66.7 (30)	0.035
		FFP2 mask	34.9 (29)	21.1 (8)	46.7 (21)	0.016
		Gown/Apron	8.4 (7)	7.9 (3)	8.9 (4)	0.88
		None	6 (5)	7.9 (3)	4.4 (2)	0.55
Protective personal hygiene measures	Distance of 1 m		87.9 (73)	86.8 (33)	88.9 (40)	0.49
	Surgical mask		54.2 (45)	73.7 (28)	44.4 (20)	0.049
	FFP2 mask		2.4 (2)	-	4.4 (2)	0.62
	None		3.6 (3)	5.3 (2)	2.2 (1)	0.24

N: number, RTPCR: reverse transcriptase polymerase chain reaction, PPE: personal protective equipment, FFP2: filtering facepiece. * Comparison between RTPCR+ and RTPCR− groups.

## Data Availability

The data presented in this study are available on request from the corresponding author. The data are not publicly available.
